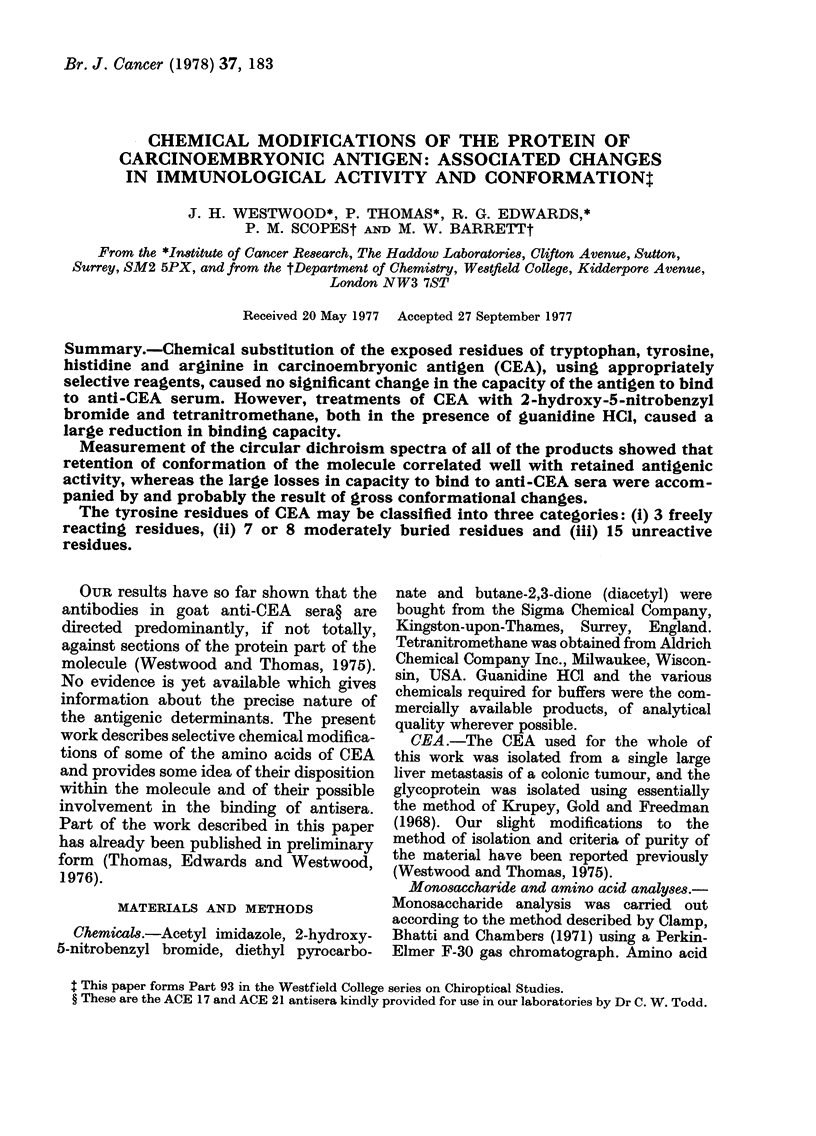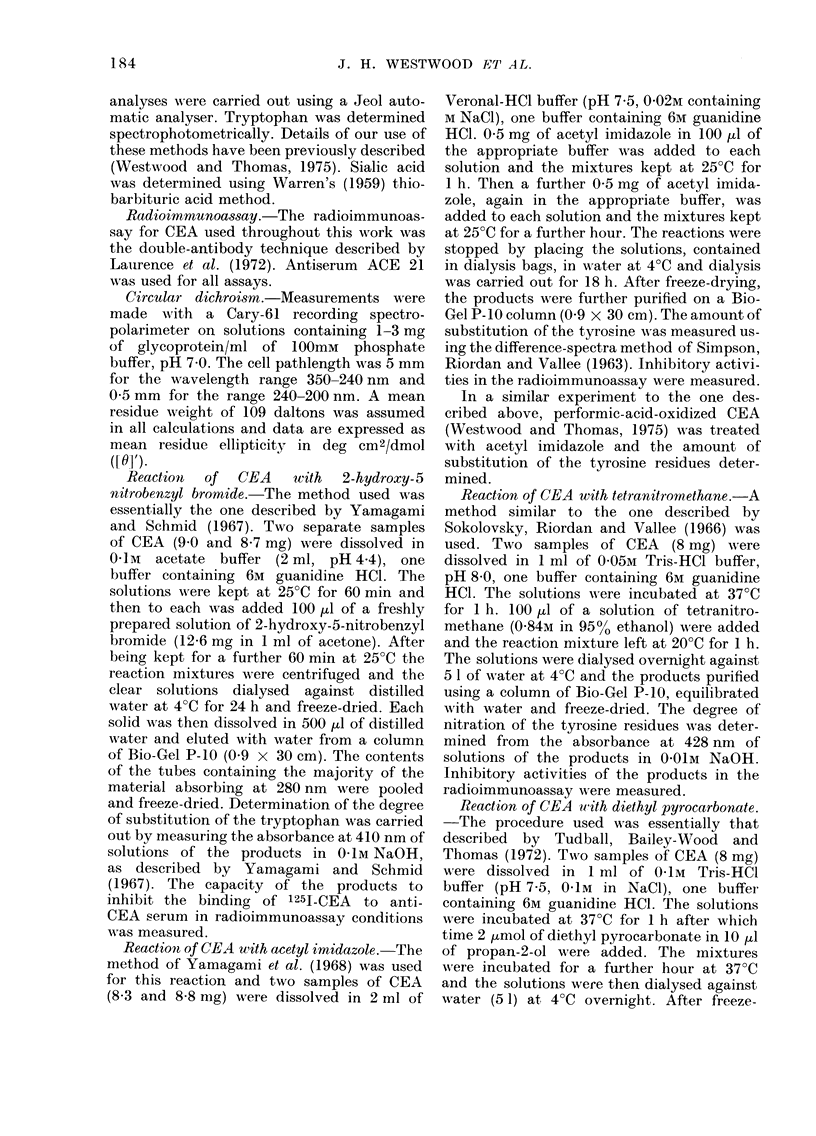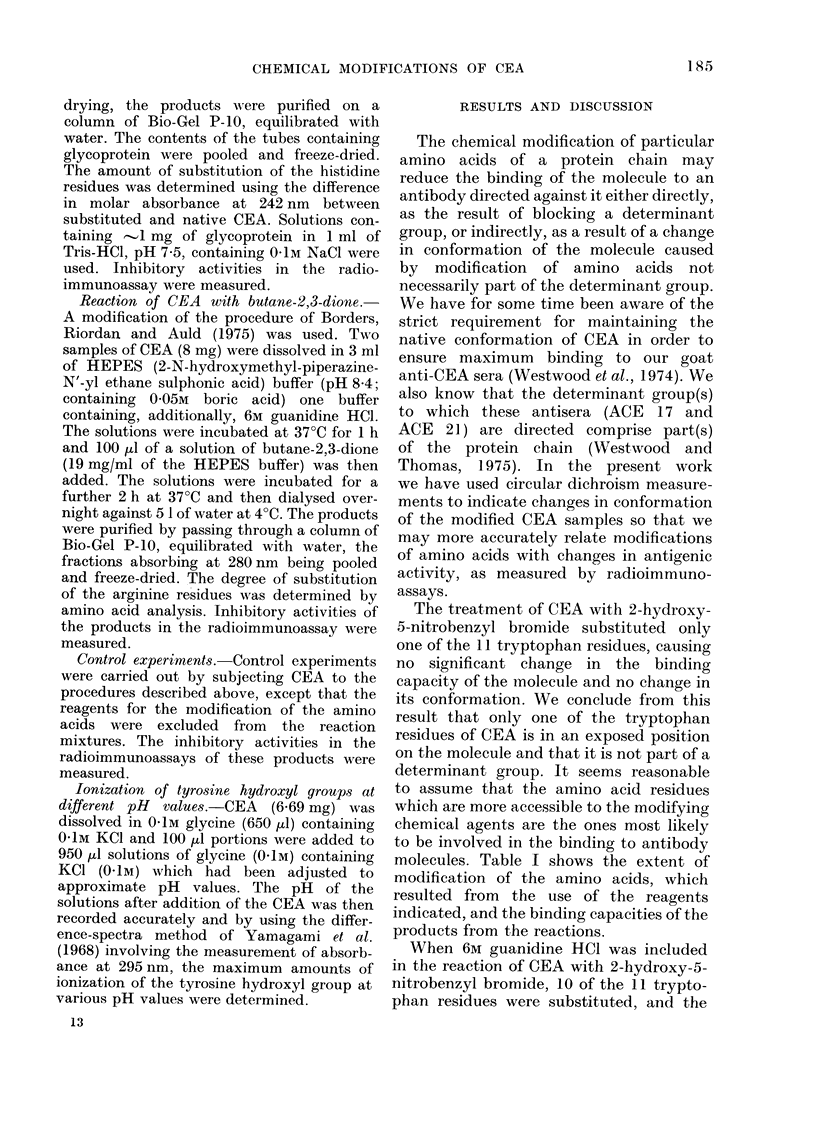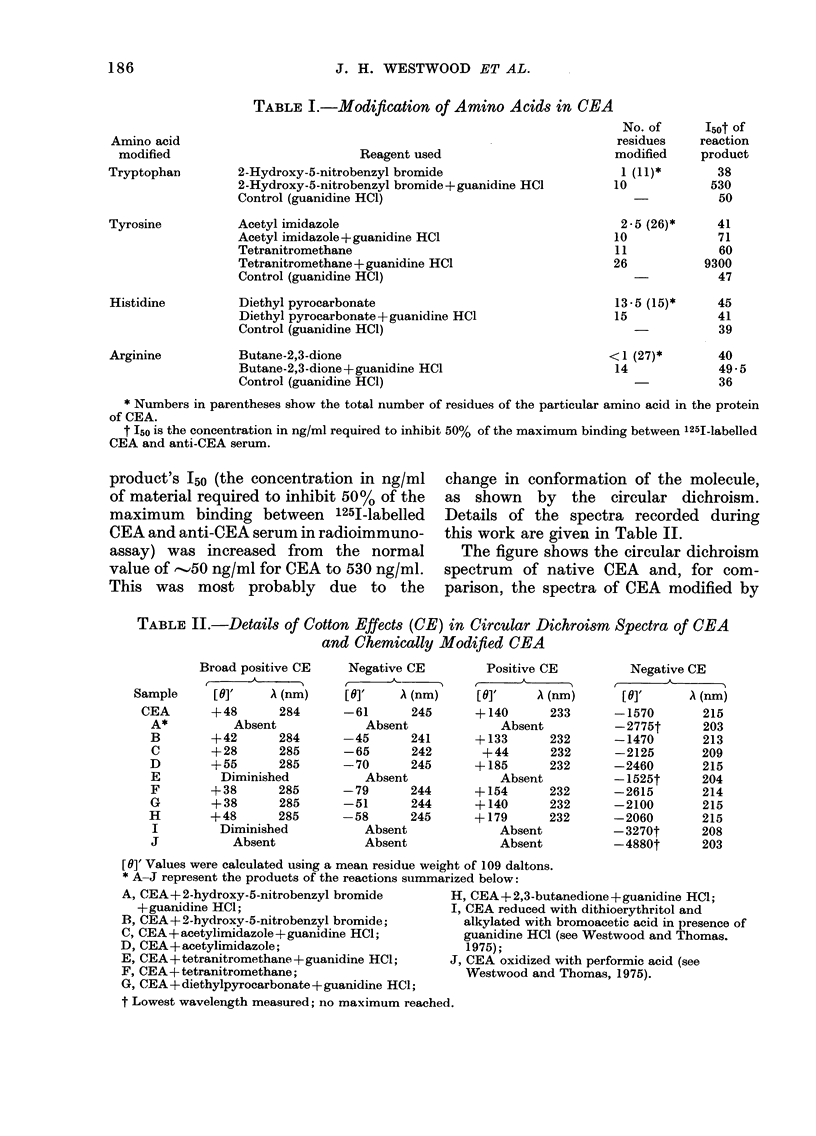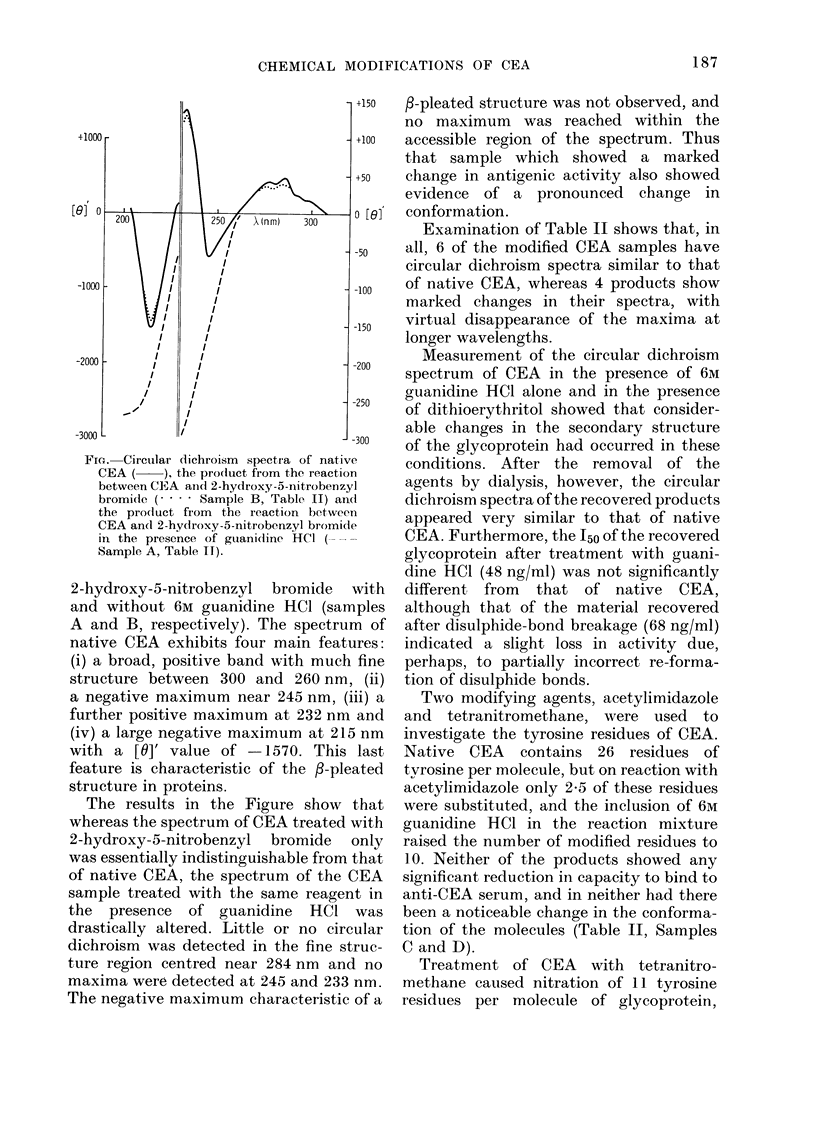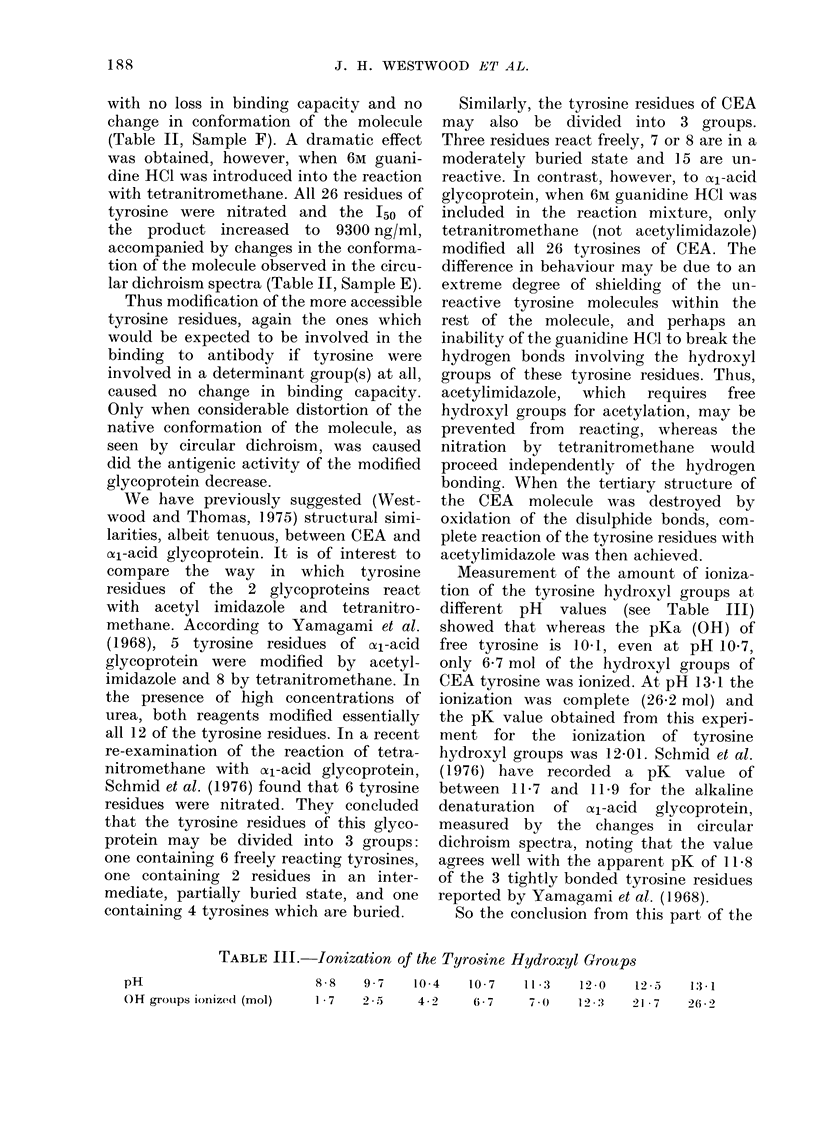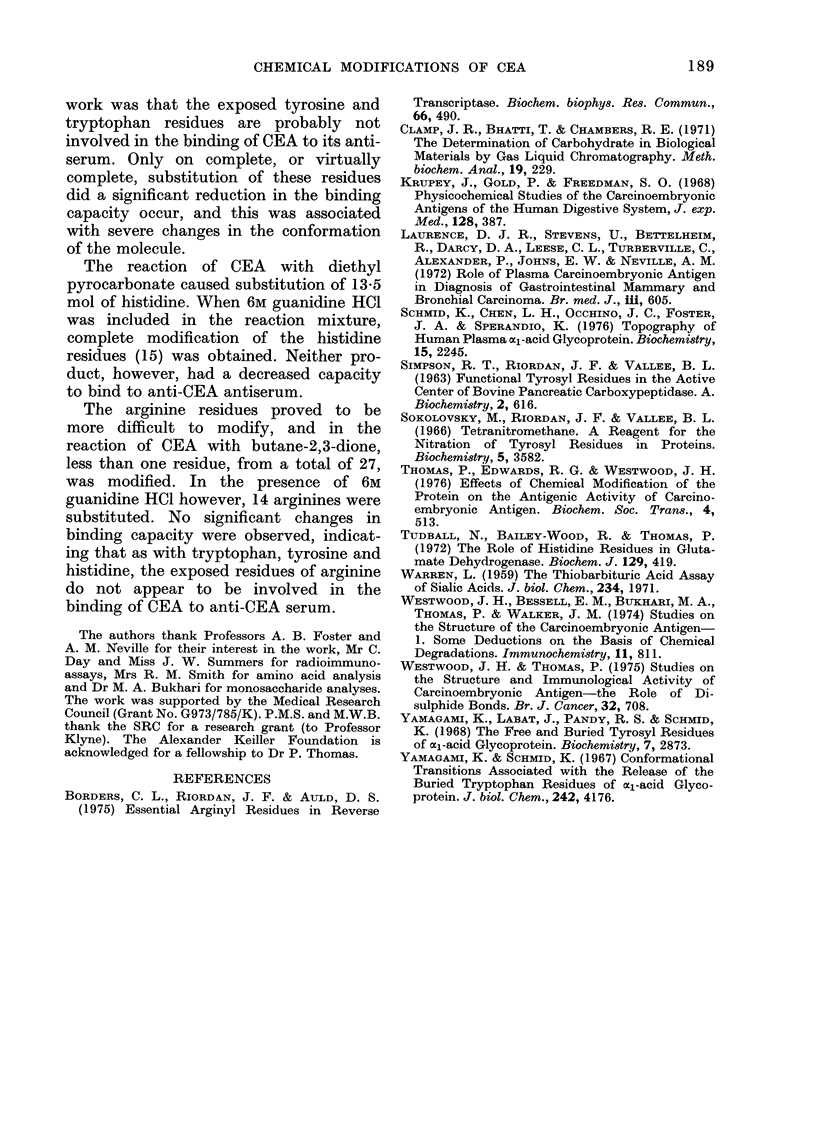# Chemical modifications of the protein of carcinoembryonic antigen: associated changes in immunological activity and conformation.

**DOI:** 10.1038/bjc.1978.26

**Published:** 1978-02

**Authors:** J. H. Westwood, P. Thomas, R. G. Edwards, P. M. Scopes, M. W. Barrett

## Abstract

Chemical substitution of the exposed residues of tryptophan, tyrosine, histidine and arginine in carcinoembryonic antigen (CEA), using appropriately selective reagents, caused no significant change in the capacity of the antigen to bind to anti-CEA serum. However, treatments of CEA with 2-hydroxy-5-nitrobenzyl bromide and tetranitromethane, both in the presence of guanidine HCl, caused a large reduction in binding capacity. Measurement of the circular dichroism spectra of all of the products showed that retention of conformation of the molecular correlated well with retained antigenic activity, whereas the large losses in capacity to bind to anti-CEA sera were accompanied by a probably the result of gross conformational changes. The tyrosine residues of CEA may be classified into three categories: (i) 3 freely reacting residues, (ii) 7 or 8 moderately buried residues and (iii) 15 unreactive residues.


Br. J. Cancer (1978) 37, 183

### CHEMICAL MODIFICATIONS OF THE PROTEIN OF CARCINOEMBRYONIC ANTIGEN: ASSOCIATED CHANGES IN IMMUNOLOGICAL ACTIVITY AND CONFORMATION‡

J. H. WESTWOOD*, P. THOMAS*, R. G. EDWARDS,* P. M. SCOPES† AND M. W. BARRETT†

*From the *Institute of Cancer Research, The Haddow Laboratories, Clifton Avenue, Sutton, Surrey, SM2 5PX, and from the †Department of Chemistry, Westfield College, Kidderpore Avenue, London NW3 7ST*

Received 20 May 1977 Accepted 27 September 1977

**Summary.—Chemical substitution of the exposed residues of tryptophan, tyrosine, histidine and arginine in carcinoembryonic antigen (CEA), using appropriately selective reagents, caused no significant change in the capacity of the antigen to bind to anti-CEA serum. However, treatments of CEA with 2-hydroxy-5-nitrobenzyl bromide and tetranitromethane, both in the presence of guanidine HCl, caused a large reduction in binding capacity.**

**Measurement of the circular dichroism spectra of all of the products showed that retention of conformation of the molecule correlated well with retained antigenic activity, whereas the large losses in capacity to bind to anti-CEA sera were accompanied by and probably the result of gross conformational changes.**

**The tyrosine residues of CEA may be classified into three categories: (i) 3 freely reacting residues, (ii) 7 or 8 moderately buried residues and (iii) 15 unreactive residues.**

OUR results have so far shown that the antibodies in goat anti-CEA sera§ are directed predominantly, if not totally, against sections of the protein part of the molecule (Westwood and Thomas, 1975). No evidence is yet available which gives information about the precise nature of the antigenic determinants. The present work describes selective chemical modifications of some of the amino acids of CEA and provides some idea of their disposition within the molecule and of their possible involvement in the binding of antisera. Part of the work described in this paper has already been published in preliminary form (Thomas, Edwards and Westwood, 1976).

### MATERIALS AND METHODS

*Chemicals.*—Acetyl imidazole, 2-hydroxy-5-nitrobenzyl bromide, diethyl pyrocarbonate and butane-2,3-dione (diacetyl) were bought from the Sigma Chemical Company, Kingston-upon-Thames, Surrey, England. Tetranitromethane was obtained from Aldrich Chemical Company Inc., Milwaukee, Wisconsin, USA. Guanidine HCl and the various chemicals required for buffers were the commercially available products, of analytical quality wherever possible.

*CEA.*—The CEA used for the whole of this work was isolated from a single large liver metastasis of a colonic tumour, and the glycoprotein was isolated using essentially the method of Krupey, Gold and Freedman (1968). Our slight modifications to the method of isolation and criteria of purity of the material have been reported previously (Westwood and Thomas, 1975).

*Monosaccharide and amino acid analyses.*—Monosaccharide analysis was carried out according to the method described by Clamp, Bhatti and Chambers (1971) using a Perkin-Elmer F-30 gas chromatograph. Amino acid

‡ This paper forms Part 93 in the Westfield College series on Chiroptical Studies.
§ These are the ACE 17 and ACE 21 antisera kindly provided for use in our laboratories by Dr C. W. Todd.

analyses were carried out using a Jeol automatic analyser. Tryptophan was determined spectrophotometrically. Details of our use of these methods have been previously described (Westwood and Thomas, 1975). Sialic acid was determined using Warren's (1959) thiobarbituric acid method.

*Radioimmunoassay.*—The radioimmunoassay for CEA used throughout this work was the double-antibody technique described by Laurence *et al.* (1972). Antiserum ACE 21 was used for all assays.

*Circular dichroism.*—Measurements were made with a Cary-61 recording spectropolarimeter on solutions containing 1–3 mg of glycoprotein/ml of 100mM phosphate buffer, pH 7·0. The cell pathlength was 5 mm for the wavelength range 350–240 nm and 0·5 mm for the range 240–200 nm. A mean residue weight of 109 daltons was assumed in all calculations and data are expressed as mean residue ellipticity in deg cm$^2$/dmol ($[\theta]'$).

*Reaction of CEA with 2-hydroxy-5 nitrobenzyl bromide.*—The method used was essentially the one described by Yamagami and Schmid (1967). Two separate samples of CEA (9·0 and 8·7 mg) were dissolved in 0·1M acetate buffer (2 ml, pH 4·4), one buffer containing 6M guanidine HCl. The solutions were kept at 25°C for 60 min and then to each was added 100 $\mu$l of a freshly prepared solution of 2-hydroxy-5-nitrobenzyl bromide (12·6 mg in 1 ml of acetone). After being kept for a further 60 min at 25°C the reaction mixtures were centrifuged and the clear solutions dialysed against distilled water at 4°C for 24 h and freeze-dried. Each solid was then dissolved in 500 $\mu$l of distilled water and eluted with water from a column of Bio-Gel P-10 (0·9 × 30 cm). The contents of the tubes containing the majority of the material absorbing at 280 nm were pooled and freeze-dried. Determination of the degree of substitution of the tryptophan was carried out by measuring the absorbance at 410 nm of solutions of the products in 0·1M NaOH, as described by Yamagami and Schmid (1967). The capacity of the products to inhibit the binding of $^{125}$I-CEA to anti-CEA serum in radioimmunoassay conditions was measured.

*Reaction of CEA with acetyl imidazole.*—The method of Yamagami *et al.* (1968) was used for this reaction and two samples of CEA (8·3 and 8·8 mg) were dissolved in 2 ml of Veronal-HCl buffer (pH 7·5, 0·02M containing M NaCl), one buffer containing 6M guanidine HCl. 0·5 mg of acetyl imidazole in 100 $\mu$l of the appropriate buffer was added to each solution and the mixtures kept at 25°C for 1 h. Then a further 0·5 mg of acetyl imidazole, again in the appropriate buffer, was added to each solution and the mixtures kept at 25°C for a further hour. The reactions were stopped by placing the solutions, contained in dialysis bags, in water at 4°C and dialysis was carried out for 18 h. After freeze-drying, the products were further purified on a Bio-Gel P-10 column (0·9 × 30 cm). The amount of substitution of the tyrosine was measured using the difference-spectra method of Simpson, Riordan and Vallee (1963). Inhibitory activities in the radioimmunoassay were measured.

In a similar experiment to the one described above, performic-acid-oxidized CEA (Westwood and Thomas, 1975) was treated with acetyl imidazole and the amount of substitution of the tyrosine residues determined.

*Reaction of CEA with tetranitromethane.*—A method similar to the one described by Sokolovsky, Riordan and Vallee (1966) was used. Two samples of CEA (8 mg) were dissolved in 1 ml of 0·05M Tris-HCl buffer, pH 8·0, one buffer containing 6M guanidine HCl. The solutions were incubated at 37°C for 1 h. 100 $\mu$l of a solution of tetranitromethane (0·84M in 95% ethanol) were added and the reaction mixture left at 20°C for 1 h. The solutions were dialysed overnight against 5 l of water at 4°C and the products purified using a column of Bio-Gel P-10, equilibrated with water and freeze-dried. The degree of nitration of the tyrosine residues was determined from the absorbance at 428 nm of solutions of the products in 0·01M NaOH. Inhibitory activities of the products in the radioimmunoassay were measured.

*Reaction of CEA with diethyl pyrocarbonate.*—The procedure used was essentially that described by Tudball, Bailey-Wood and Thomas (1972). Two samples of CEA (8 mg) were dissolved in 1 ml of 0·1M Tris-HCl buffer (pH 7·5, 0·1M in NaCl), one buffer containing 6M guanidine HCl. The solutions were incubated at 37°C for 1 h after which time 2 $\mu$mol of diethyl pyrocarbonate in 10 $\mu$l of propan-2-ol were added. The mixtures were incubated for a further hour at 37°C and the solutions were then dialysed against water (5 l) at 4°C overnight. After freezedrying, the products were purified on a column of Bio-Gel P-10, equilibrated with water. The contents of the tubes containing glycoprotein were pooled and freeze-dried. The amount of substitution of the histidine residues was determined using the difference in molar absorbance at 242 nm between substituted and native CEA. Solutions containing ~1 mg of glycoprotein in 1 ml of Tris-HCl, pH 7·5, containing 0·1M NaCl were used. Inhibitory activities in the radioimmunoassay were measured.

*Reaction of CEA with butane-2,3-dione.*—A modification of the procedure of Borders, Riordan and Auld (1975) was used. Two samples of CEA (8 mg) were dissolved in 3 ml of HEPES (2-N-hydroxymethyl-piperazine-N′-yl ethane sulphonic acid) buffer (pH 8·4; containing 0·05M boric acid) one buffer containing, additionally, 6M guanidine HCl. The solutions were incubated at 37°C for 1 h and 100 μl of a solution of butane-2,3-dione (19 mg/ml of the HEPES buffer) was then added. The solutions were incubated for a further 2 h at 37°C and then dialysed overnight against 5 l of water at 4°C. The products were purified by passing through a column of Bio-Gel P-10, equilibrated with water, the fractions absorbing at 280 nm being pooled and freeze-dried. The degree of substitution of the arginine residues was determined by amino acid analysis. Inhibitory activities of the products in the radioimmunoassay were measured.

*Control experiments.*—Control experiments were carried out by subjecting CEA to the procedures described above, except that the reagents for the modification of the amino acids were excluded from the reaction mixtures. The inhibitory activities in the radioimmunoassays of these products were measured.

*Ionization of tyrosine hydroxyl groups at different pH values.*—CEA (6·69 mg) was dissolved in 0·1M glycine (650 μl) containing 0·1M KCl and 100 μl portions were added to 950 μl solutions of glycine (0·1M) containing KCl (0·1M) which had been adjusted to approximate pH values. The pH of the solutions after addition of the CEA was then recorded accurately and by using the difference-spectra method of Yamagami *et al.* (1968) involving the measurement of absorbance at 295 nm, the maximum amounts of ionization of the tyrosine hydroxyl group at various pH values were determined.

### RESULTS AND DISCUSSION

The chemical modification of particular amino acids of a protein chain may reduce the binding of the molecule to an antibody directed against it either directly, as the result of blocking a determinant group, or indirectly, as a result of a change in conformation of the molecule caused by modification of amino acids not necessarily part of the determinant group. We have for some time been aware of the strict requirement for maintaining the native conformation of CEA in order to ensure maximum binding to our goat anti-CEA sera (Westwood *et al.*, 1974). We also know that the determinant group(s) to which these antisera (ACE 17 and ACE 21) are directed comprise part(s) of the protein chain (Westwood and Thomas, 1975). In the present work we have used circular dichroism measurements to indicate changes in conformation of the modified CEA samples so that we may more accurately relate modifications of amino acids with changes in antigenic activity, as measured by radioimmunoassays.

The treatment of CEA with 2-hydroxy-5-nitrobenzyl bromide substituted only one of the 11 tryptophan residues, causing no significant change in the binding capacity of the molecule and no change in its conformation. We conclude from this result that only one of the tryptophan residues of CEA is in an exposed position on the molecule and that it is not part of a determinant group. It seems reasonable to assume that the amino acid residues which are more accessible to the modifying chemical agents are the ones most likely to be involved in the binding to antibody molecules. Table I shows the extent of modification of the amino acids, which resulted from the use of the reagents indicated, and the binding capacities of the products from the reactions.

When 6M guanidine HCl was included in the reaction of CEA with 2-hydroxy-5-nitrobenzyl bromide, 10 of the 11 tryptophan residues were substituted, and the

TABLE I.—*Modification of Amino Acids in CEA*

| Amino acid modified | Reagent used | No. of residues modified | $I_{50}$† of reaction product |
|---|---|---|---|
| Tryptophan | 2-Hydroxy-5-nitrobenzyl bromide | 1 (11)* | 38 |
| | 2-Hydroxy-5-nitrobenzyl bromide + guanidine HCl | 10 | 530 |
| | Control (guanidine HCl) | — | 50 |
| Tyrosine | Acetyl imidazole | 2·5 (26)* | 41 |
| | Acetyl imidazole + guanidine HCl | 10 | 71 |
| | Tetranitromethane | 11 | 60 |
| | Tetranitromethane + guanidine HCl | 26 | 9300 |
| | Control (guanidine HCl) | — | 47 |
| Histidine | Diethyl pyrocarbonate | 13·5 (15)* | 45 |
| | Diethyl pyrocarbonate + guanidine HCl | 15 | 41 |
| | Control (guanidine HCl) | — | 39 |
| Arginine | Butane-2,3-dione | <1 (27)* | 40 |
| | Butane-2,3-dione + guanidine HCl | 14 | 49·5 |
| | Control (guanidine HCl) | — | 36 |

* Numbers in parentheses show the total number of residues of the particular amino acid in the protein of CEA.

† $I_{50}$ is the concentration in ng/ml required to inhibit 50% of the maximum binding between $^{125}I$-labelled CEA and anti-CEA serum.

product's $I_{50}$ (the concentration in ng/ml of material required to inhibit 50% of the maximum binding between $^{125}I$-labelled CEA and anti-CEA serum in radioimmunoassay) was increased from the normal value of ~50 ng/ml for CEA to 530 ng/ml. This was most probably due to the change in conformation of the molecule, as shown by the circular dichroism. Details of the spectra recorded during this work are given in Table II.

The figure shows the circular dichroism spectrum of native CEA and, for comparison, the spectra of CEA modified by

TABLE II.—*Details of Cotton Effects (CE) in Circular Dichroism Spectra of CEA and Chemically Modified CEA*

| Sample | Broad positive CE | | Negative CE | | Positive CE | | Negative CE | |
|---|---|---|---|---|---|---|---|---|
| | $[\theta]'$ | λ (nm) | $[\theta]'$ | λ (nm) | $[\theta]'$ | λ (nm) | $[\theta]'$ | λ (nm) |
| CEA | +48 | 284 | −61 | 245 | +140 | 233 | −1570 | 215 |
| A* | Absent | | Absent | | Absent | | −2775† | 203 |
| B | +42 | 284 | −45 | 241 | +133 | 232 | −1470 | 213 |
| C | +28 | 285 | −65 | 242 | +44 | 232 | −2125 | 209 |
| D | +55 | 285 | −70 | 245 | +185 | 232 | −2460 | 215 |
| E | Diminished | | Absent | | Absent | | −1525† | 204 |
| F | +38 | 285 | −79 | 244 | +154 | 232 | −2615 | 214 |
| G | +38 | 285 | −51 | 244 | +140 | 232 | −2100 | 215 |
| H | +48 | 285 | −58 | 245 | +179 | 232 | −2060 | 215 |
| I | Diminished | | Absent | | Absent | | −3270† | 208 |
| J | Absent | | Absent | | Absent | | −4880† | 203 |

$[\theta]'$ Values were calculated using a mean residue weight of 109 daltons.

* A–J represent the products of the reactions summarized below:

A, CEA + 2-hydroxy-5-nitrobenzyl bromide + guanidine HCl;
B, CEA + 2-hydroxy-5-nitrobenzyl bromide;
C, CEA + acetylimidazole + guanidine HCl;
D, CEA + acetylimidazole;
E, CEA + tetranitromethane + guanidine HCl;
F, CEA + tetranitromethane;
G, CEA + diethylpyrocarbonate + guanidine HCl;
H, CEA + 2,3-butanedione + guanidine HCl;
I, CEA reduced with dithioerythritol and alkylated with bromoacetic acid in presence of guanidine HCl (see Westwood and Thomas. 1975);
J, CEA oxidized with performic acid (see Westwood and Thomas, 1975).

† Lowest wavelength measured; no maximum reached.

FIG.—Circular dichroism spectra of native CEA (——), the product from the reaction between CEA and 2-hydroxy-5-nitrobenzyl bromide (· · · · Sample B, Table II) and the product from the reaction between CEA and 2-hydroxy-5-nitrobenzyl bromide in the presence of guanidine HCl (– – – Sample A, Table II).

2-hydroxy-5-nitrobenzyl bromide with and without 6M guanidine HCl (samples A and B, respectively). The spectrum of native CEA exhibits four main features: (i) a broad, positive band with much fine structure between 300 and 260 nm, (ii) a negative maximum near 245 nm, (iii) a further positive maximum at 232 nm and (iv) a large negative maximum at 215 nm with a $[\theta]'$ value of $-1570$. This last feature is characteristic of the $\beta$-pleated structure in proteins.

The results in the Figure show that whereas the spectrum of CEA treated with 2-hydroxy-5-nitrobenzyl bromide only was essentially indistinguishable from that of native CEA, the spectrum of the CEA sample treated with the same reagent in the presence of guanidine HCl was drastically altered. Little or no circular dichroism was detected in the fine structure region centred near 284 nm and no maxima were detected at 245 and 233 nm. The negative maximum characteristic of a $\beta$-pleated structure was not observed, and no maximum was reached within the accessible region of the spectrum. Thus that sample which showed a marked change in antigenic activity also showed evidence of a pronounced change in conformation.

Examination of Table II shows that, in all, 6 of the modified CEA samples have circular dichroism spectra similar to that of native CEA, whereas 4 products show marked changes in their spectra, with virtual disappearance of the maxima at longer wavelengths.

Measurement of the circular dichroism spectrum of CEA in the presence of 6M guanidine HCl alone and in the presence of dithioerythritol showed that considerable changes in the secondary structure of the glycoprotein had occurred in these conditions. After the removal of the agents by dialysis, however, the circular dichroism spectra of the recovered products appeared very similar to that of native CEA. Furthermore, the $I_{50}$ of the recovered glycoprotein after treatment with guanidine HCl (48 ng/ml) was not significantly different from that of native CEA, although that of the material recovered after disulphide-bond breakage (68 ng/ml) indicated a slight loss in activity due, perhaps, to partially incorrect re-formation of disulphide bonds.

Two modifying agents, acetylimidazole and tetranitromethane, were used to investigate the tyrosine residues of CEA. Native CEA contains 26 residues of tyrosine per molecule, but on reaction with acetylimidazole only 2·5 of these residues were substituted, and the inclusion of 6M guanidine HCl in the reaction mixture raised the number of modified residues to 10. Neither of the products showed any significant reduction in capacity to bind to anti-CEA serum, and in neither had there been a noticeable change in the conformation of the molecules (Table II, Samples C and D).

Treatment of CEA with tetranitromethane caused nitration of 11 tyrosine residues per molecule of glycoprotein, with no loss in binding capacity and no change in conformation of the molecule (Table II, Sample F). A dramatic effect was obtained, however, when 6M guanidine HCl was introduced into the reaction with tetranitromethane. All 26 residues of tyrosine were nitrated and the $I_{50}$ of the product increased to 9300 ng/ml, accompanied by changes in the conformation of the molecule observed in the circular dichroism spectra (Table II, Sample E).

Thus modification of the more accessible tyrosine residues, again the ones which would be expected to be involved in the binding to antibody if tyrosine were involved in a determinant group(s) at all, caused no change in binding capacity. Only when considerable distortion of the native conformation of the molecule, as seen by circular dichroism, was caused did the antigenic activity of the modified glycoprotein decrease.

We have previously suggested (Westwood and Thomas, 1975) structural similarities, albeit tenuous, between CEA and $\alpha_1$-acid glycoprotein. It is of interest to compare the way in which tyrosine residues of the 2 glycoproteins react with acetyl imidazole and tetranitromethane. According to Yamagami *et al.* (1968), 5 tyrosine residues of $\alpha_1$-acid glycoprotein were modified by acetylimidazole and 8 by tetranitromethane. In the presence of high concentrations of urea, both reagents modified essentially all 12 of the tyrosine residues. In a recent re-examination of the reaction of tetranitromethane with $\alpha_1$-acid glycoprotein, Schmid *et al.* (1976) found that 6 tyrosine residues were nitrated. They concluded that the tyrosine residues of this glycoprotein may be divided into 3 groups: one containing 6 freely reacting tyrosines, one containing 2 residues in an intermediate, partially buried state, and one containing 4 tyrosines which are buried.

Similarly, the tyrosine residues of CEA may also be divided into 3 groups. Three residues react freely, 7 or 8 are in a moderately buried state and 15 are unreactive. In contrast, however, to $\alpha_1$-acid glycoprotein, when 6M guanidine HCl was included in the reaction mixture, only tetranitromethane (not acetylimidazole) modified all 26 tyrosines of CEA. The difference in behaviour may be due to an extreme degree of shielding of the unreactive tyrosine molecules within the rest of the molecule, and perhaps an inability of the guanidine HCl to break the hydrogen bonds involving the hydroxyl groups of these tyrosine residues. Thus, acetylimidazole, which requires free hydroxyl groups for acetylation, may be prevented from reacting, whereas the nitration by tetranitromethane would proceed independently of the hydrogen bonding. When the tertiary structure of the CEA molecule was destroyed by oxidation of the disulphide bonds, complete reaction of the tyrosine residues with acetylimidazole was then achieved.

Measurement of the amount of ionization of the tyrosine hydroxyl groups at different pH values (see Table III) showed that whereas the pKa (OH) of free tyrosine is 10·1, even at pH 10·7, only 6·7 mol of the hydroxyl groups of CEA tyrosine was ionized. At pH 13·1 the ionization was complete (26·2 mol) and the pK value obtained from this experiment for the ionization of tyrosine hydroxyl groups was 12·01. Schmid *et al.* (1976) have recorded a pK value of between 11·7 and 11·9 for the alkaline denaturation of $\alpha_1$-acid glycoprotein, measured by the changes in circular dichroism spectra, noting that the value agrees well with the apparent pK of 11·8 of the 3 tightly bonded tyrosine residues reported by Yamagami *et al.* (1968).

So the conclusion from this part of the

TABLE III.—*Ionization of the Tyrosine Hydroxyl Groups*

| pH | 8·8 | 9·7 | 10·4 | 10·7 | 11·3 | 12·0 | 12·5 | 13·1 |
|---|---|---|---|---|---|---|---|---|
| OH groups ionized (mol) | 1·7 | 2·5 | 4·2 | 6·7 | 7·0 | 12·3 | 21·7 | 26·2 |

work was that the exposed tyrosine and tryptophan residues are probably not involved in the binding of CEA to its antiserum. Only on complete, or virtually complete, substitution of these residues did a significant reduction in the binding capacity occur, and this was associated with severe changes in the conformation of the molecule.

The reaction of CEA with diethyl pyrocarbonate caused substitution of 13·5 mol of histidine. When 6M guanidine HCl was included in the reaction mixture, complete modification of the histidine residues (15) was obtained. Neither product, however, had a decreased capacity to bind to anti-CEA antiserum.

The arginine residues proved to be more difficult to modify, and in the reaction of CEA with butane-2,3-dione, less than one residue, from a total of 27, was modified. In the presence of 6M guanidine HCl however, 14 arginines were substituted. No significant changes in binding capacity were observed, indicating that as with tryptophan, tyrosine and histidine, the exposed residues of arginine do not appear to be involved in the binding of CEA to anti-CEA serum.

The authors thank Professors A. B. Foster and A. M. Neville for their interest in the work, Mr C. Day and Miss J. W. Summers for radioimmunoassays, Mrs R. M. Smith for amino acid analysis and Dr M. A. Bukhari for monosaccharide analyses. The work was supported by the Medical Research Council (Grant No. G973/785/K). P.M.S. and M.W.B. thank the SRC for a research grant (to Professor Klyne). The Alexander Keiller Foundation is acknowledged for a fellowship to Dr P. Thomas.

### REFERENCES

Borders, C. L., Riordan, J. F. & Auld, D. S. (1975) Essential Arginyl Residues in Reverse Transcriptase. *Biochem. biophys. Res. Commun.*, **66**, 490.

Clamp, J. R., Bhatti, T. & Chambers, R. E. (1971) The Determination of Carbohydrate in Biological Materials by Gas Liquid Chromatography. *Meth. biochem. Anal.*, **19**, 229.

Krupey, J., Gold, P. & Freedman, S. O. (1968) Physicochemical Studies of the Carcinoembryonic Antigens of the Human Digestive System, *J. exp. Med.*, **128**, 387.

Laurence, D. J. R., Stevens, U., Bettelheim, R., Darcy, D. A., Leese, C. L., Turberville, C., Alexander, P., Johns, E. W. & Neville, A. M. (1972) Role of Plasma Carcinoembryonic Antigen in Diagnosis of Gastrointestinal Mammary and Bronchial Carcinoma. *Br. med. J.*, **iii**, 605.

Schmid, K., Chen, L. H., Occhino, J. C., Foster, J. A. & Sperandio, K. (1976) Topography of Human Plasma $\alpha_1$-acid Glycoprotein. *Biochemistry*, **15**, 2245.

Simpson, R. T., Riordan, J. F. & Vallee, B. L. (1963) Functional Tyrosyl Residues in the Active Center of Bovine Pancreatic Carboxypeptidase. A. *Biochemistry*, **2**, 616.

Sokolovsky, M., Riordan, J. F. & Vallee, B. L. (1966) Tetranitromethane. A Reagent for the Nitration of Tyrosyl Residues in Proteins. *Biochemistry*, **5**, 3582.

Thomas, P., Edwards, R. G. & Westwood, J. H. (1976) Effects of Chemical Modification of the Protein on the Antigenic Activity of Carcinoembryonic Antigen. *Biochem. Soc. Trans.*, **4**, 513.

Tudball, N., Bailey-Wood, R. & Thomas, P. (1972) The Role of Histidine Residues in Glutamate Dehydrogenase. *Biochem. J.* **129**, 419.

Warren, L. (1959) The Thiobarbituric Acid Assay of Sialic Acids. *J. biol. Chem.*, **234**, 1971.

Westwood, J. H., Bessell, E. M., Bukhari, M. A., Thomas, P. & Walker, J. M. (1974) Studies on the Structure of the Carcinoembryonic Antigen—1. Some Deductions on the Basis of Chemical Degradations. *Immunochemistry*, **11**, 811.

Westwood, J. H. & Thomas, P. (1975) Studies on the Structure and Immunological Activity of Carcinoembryonic Antigen—the Role of Disulphide Bonds. *Br. J. Cancer*, **32**, 708.

Yamagami, K., Labat, J., Pandy, R. S. & Schmid, K. (1968) The Free and Buried Tyrosyl Residues of $\alpha_1$-acid Glycoprotein. *Biochemistry*, **7**, 2873.

Yamagami, K. & Schmid, K. (1967) Conformational Transitions Associated with the Release of the Buried Tryptophan Residues of $\alpha_1$-acid Glycoprotein. *J. biol. Chem.*, **242**, 4176.